# Biophysical characterization of the first ultra-cyclist in the world to break the 1,000 km barrier in 24-h non-stop road cycling: A case report

**DOI:** 10.3389/fcvm.2022.990382

**Published:** 2022-10-11

**Authors:** Beat Knechtle, Pedro Forte, Katja Weiss, Ivan Cuk, Pantelis T. Nikolaidis, Caio Victor Sousa, Marilia Santos Andrade, Mabliny Thuany

**Affiliations:** ^1^Medbase St. Gallen Am Vadianplatz, St. Gallen, Switzerland; ^2^Institute of Primary Care, University of Zurich, Zurich, Switzerland; ^3^Higher Institute of Educational Sciences of the Douro, Penafiel, Portugal; ^4^Instituto Politécnico de Bragança, Bragança, Portugal; ^5^Research Center in Sports, Health and Human Development, Covilhã, Portugal; ^6^Faculty of Sport and Physical Education, University of Belgrade, Belgrade, Serbia; ^7^School of Health and Caring Sciences, University of West Attica, Athens, Greece; ^8^Health and Human Sciences, Loyola Marymount University, Los Angeles, CA, United States; ^9^Departamento de Fisiologia, Disciplina de Neurofisiologia e Fisiologia do Exercício, Universidade Federal de São Paulo, São Paulo, Brazil; ^10^Centre of Research, Education, Innovation and Intervention in Sport (CIFI2D), Faculty of Sport, University of Porto, Porto, Portugal

**Keywords:** case report, ultra-cycling, performance, aerodynamics, mechanical power

## Abstract

A plethora of factors determine elite cycling performance. Those include training characteristics, pacing strategy, aerodynamics, nutritional habits, psychological traits, physical fitness level, body mass composition, and contextual features; even the slightest changes in any of these factors can be associated with performance improvement or deterioration. The aim of the present case report is to compare the performances of the same ultra-cyclist in achieving two world records (WR) in 24 h cycling. We have analyzed and compared the distance covered and speed for each WR. The 24 h period was split into four-time intervals (0–6 h; > 6–12 h; > 12–18 h; > 18–24 h), and we compared the differences in the distance covered and speed between the two WRs. For both WRs, a strong negative correlation between distance and speed was confirmed (*r* = –0.85; *r* = –0.89, for old and new WR, respectively). Differences in speed (km/h) were shown between the two WRs, with the most significant differences in 12–18 h (Δ = 6.50 km/h). For the covered distance in each block, the most significant differences were observed in the last part of the cycling (Δ = 38.54 km). The cyclist effective surface area (ACd) was 0.25 m^2^ less and 20% more drag in the new WR. Additionally, the mechanical power was 8%, the power to overcome drag was 31%, and the power-weight ratio was 8% higher in the new WR. The mechanical efficiency of the cyclist was 1% higher in the new WR. Finally, the heart rate (HR) presented significant differences for the first 6 h (Old WR: 145.80 ± 5.88 bpm; New WR: 139.45 ± 5.82 bpm) and between the 12 and 18 h time interval (Old WR: 133.19 ± 3.53 bpm; New WR: 137.63 ± 2.80 bpm). The marginal gains concept can explain the performance improvement in the new WR, given that the athlete made some improvements in technical specifications after the old WR.

## Introduction

Ultra-endurance events are of increasing popularity (1), with ultra-cycling attracting more and more participants in time-limited (2) and distance-limited (3, 4) events. In ultra-cycling, finishing the longest non-stop race, the “Race Across America” (RAAM), is one of the ultimate goals (5). The cyclists, coaches and analysts try to explain and control as much as possible the different variables that may affect performance (i.e., arrival time). The environmental conditions (such as temperature and humidity) (6), place of events and altitude (7), individual characteristics (i.e., anthropometrics) (8), biomechanical issues (i.e., interaction between cyclist and bicycle) (9), physical and physiological demands, altogether may explain the cyclist winning time (10).

However, another important achievement in ultra-cycling is breaking the barrier of 1,000 km in a 24-h self-paced time trial. Christoph Strasser, one of the best ultra-cyclists in history and six times winner of the RAAM, set in 2015 a new world record (WR) in 24-h non-drafting road cycling with 896.173 km (11). In October 2020, Stanislav Verstovšek broke that record by achieving 914.020 km.^1^ In July 2021, Christoph Strasser broke the 24-h self-paced time trial WR again and was the first ultra-cyclist ever to break the 1,000 km barrier in 24 h.^2^

The cyclist’s performance depends on the capacity to generate sufficient propulsion to overcome the resistance acting on a cyclist (external forces) (12). Those external forces mainly consist of the drag and rolling resistance, where the drag represents about 90% of the total resistance to overcome (13–15). That highlights the importance of aerodynamics in cycling regarding performance. The cyclist has to deliver sufficient mechanical power to overcome the resistance and increase the velocity (16). Additionally, the velocity results from the quadratic ratio between the kinetic energy and the mass (Equation 1).


(1) $$v=\sqrt{\frac{2.\varepsilon_{k\,i\,n}}{m}}$$

Based on Equation 1, Equation 2 is possible to obtain:


(2) $$v=\sqrt{\frac{2(\varepsilon_{i\,n}-\varepsilon_{loss)}}{m}}$$

Where ε_*kin*_ is the energy lost (ε_*loss*_) subtracted from the energy delivered by the cyclist (ε_*in*_). Assessing the energy cost will allow us to evaluate the cyclist’s effort and reach efficiency for a selected velocity or pace (17, 18).

The physiological demands of cycling depend on various factors. The heart rate (HR) is a valid and accurate variable to quantify exercise load and intensity, especially in different competition settings in the professional cycling (19, 20). The HR analysis also monitors the session load and quantifies the physiological demands (19, 21, 22). Therefore, the HR analysis between competitions provides a better understanding of the load and intensity of the exercise and racing strategies (19, 20, 23).

In this way, we believe that information provided in the present study can be used to offer insights for athletes and coaches who focus on improving performance. In the present case study, we compare the pacing, aerodynamics, mechanical power, efficiency and physiological demands (i.e., HR) in the two WR (2015 and 2021) achieved by the same ultra-cyclist.

## Case description

### The athlete

Our subject is a professional ultra-cyclist who won the RAAM six times.^3^ Among road-based races, he also set the WR in time-limited self-paced events. In 2015, he set a new WR in the 24-h road cycling (11), and in 2017 he set another one in the 24-h track cycling (24).

In the 365 days before the first WR, he invested 1,093 h of training at a TSS (Training Stress Score) of 44,300. Before the event, he had a 78 kg body mass and 1.86 m body height. In the 365 days before the second WR, he increased training to 1,101 h at a TSS of 44,345. In contrast to the first WR, the preparation for the second WR included more polarized training, intervals of higher intensity, and basic training at lower intensities.

### The event

In 2021, he tried to break the WR in 24-h road cycling again. From July 16, 05:00 p.m. to July 17, 2021, 05:00 p.m., he finished 136 laps on a 7.58 km course at ‘‘Fliegerhorst Zeltweg’’^4^ to achieve a total distance of 1026.215 km at an average cycling speed of 42.75 km/h.^5^ The course lies at 670 m above sea level, the average temperature was constant at 15°C, the sky was covered, and rain was falling sporadically with 9 h of rainfall during his attempt. One break of 2 min had to be made to change the bike and clothes. He had two flat tires, the first shortly after midnight and the second just before finishing. The same equipment as in the previous attempt was used with some changes and improvements such as eliminating the bottle holder, improving the aerodynamic position by elevating the handlebar and optimizing the stream-lined position of the upper body, and wearing a special time-trial dress and helmet for time-trial cycling, using a larger front chain wheel (from 50 to 58) with another bottom bracket, ceramic wheel bearings, and tires of 26 (see text footnote 5).^6^^,^^7^

During the record attempt, he consumed a total of 13,452 kcal in the form of Peeroton Hi-End Endurance (17,5 bottles of 750 ml),^8^ Ensure Plus (one bottle of 200 ml before the start, then one bottle hourly during the event)^9^ and hydrogels toward the end (three gels of 60 ml). Overall, he consumed 775 ml of fluids and 100 g of carbohydrates hourly. In addition, he consumed Panaceo Energy Boost.^10^ Power in W was continuously measured using a power meter.^11^

**FIGURE 1:**
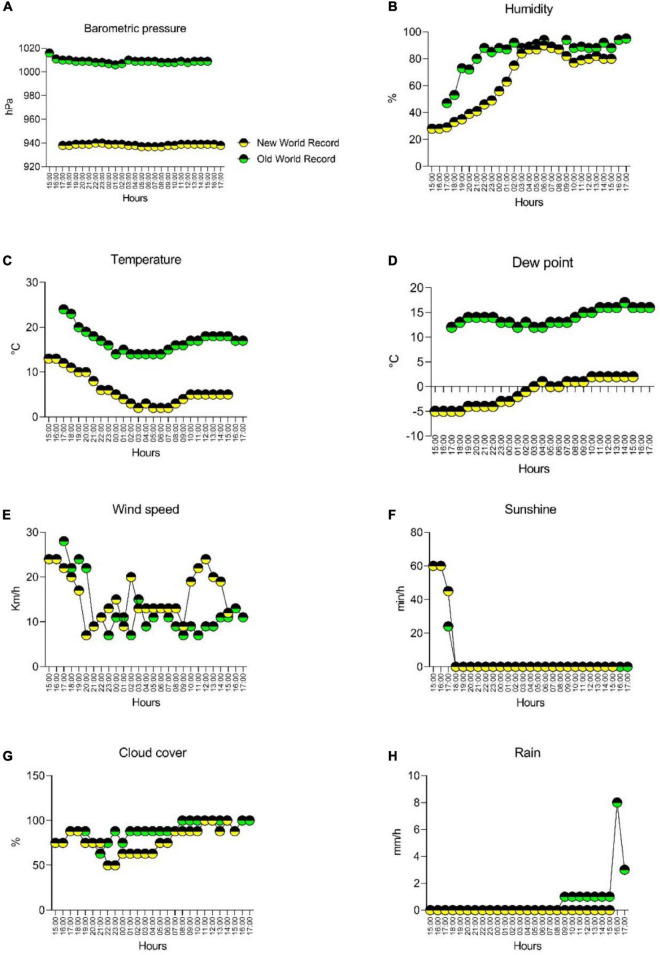
Hourly weather data for barometric pressure **(A)**, humidity **(B)**, temperature **(C)**, dew point **(D)**, wind speed **(E)**, sunshine **(F)**, cloud cover **(G)**, and rain **(H)** for both records.

Table 1 summarizes specific details of the two WRs, such as environmental conditions, energy expenditure, altitude, temperature, performance, etc. All details were provided by the athlete. Figure 1 presents the hourly weather data for barometric pressure, humidity, temperature, dew point, wind speed, sunshine, cloud cover and rain for both WR. Hourly weather data were obtained from a weather archive: https://kachelmannwetter.com/ch/messwerte/murtal for the WR in 2021 in Zeltweg, Austria (new WR) and from https://kachelmannwetter.com/ch/messwerte/stadt-berlin for the WR in 2015 in Berlin (old WR), Germany.

**TABLE 1:** Details (performance characteristics, energy expenditure, environmental conditions, altitude, temperature) of the three world records.

	1 day 1,000 k, Zeltweg	24 h road, Berlin
Category	24 h road	24 h road
Location	Fliegerhorst Hinterstoisser, Zeltweg, AUT	Tempelhofer Park, Berlin, GER
Starting date	July 16, 2021, 05:00 p.m.	March 20, 2015, 03:00 p.m.
Time	24:00:00 h	24:00:00 h
Distance	1,026.2 km	896.17 km
Elevation gain	2,601 m	1,650 m
Average speed	42,75 km/h	37,34 km/h
Normalized power	275 W	254 W
TSS training stress score	1,220	1,050
Average heart rate	136 bpm	137 bpm
Fluid intake	18,5 L	15,2 L
Energy intake	13,452 kcal	12,740 kcal
Energy consumption	23,500 kcal + ca. 4,000 kcal basal metabolic rate	21,900 kcal + ca. 4,000 kcal basal metabolic rate
Average pedaling frequency	78 U/min.	80 U/min
Lap length	7,58 km	11,73 km
Number of laps	136	77
Meters above mean sea level	670 m MSL	50 m MSL
Temperature	15°C	3°C
Humidity	84.76% ( ± 11.90)	64.20% ( ± 22.87)
Density of air (Kg/m^3^)	1.22	1.27
Top speed	61,6 km/h	49,2 km/h
Bodyweight	80 kg	78 kg
Total mechanical power	275 W	254 W
Power per weight ratio	3,44 W/kg	3,25 W/kg
Power to overcome the drag	225.68 W	156.64 W
Gross mechanical efficiency	21%	20%
Cyclist effective surface area	0.221 m^2^	0.220 m^2^
Total drag at the mean speed	15.10 N	18.99 N
Standing time	2 min	7 min
Breakdown	2 patching	1 patching

### Aerodynamics

Considering the weather conditions and the participant anthropometrics, it is possible to estimate the drag for the cyclist mean velocity. The drag is given by Equation 1.


(3) $$F_{d}=0.5\,p\,A\,C_{d}\,v^{2}$$

Where, p is the air density, A is the surface area, and Cd is the drag coefficient. The online calculator proposed by Czernia and Szyk (25) was used to assess the air density.

The A and Cd are possible to obtain based on the cyclist’s anthropometrics (Equations 2 and 3, respectively).


(4) $$A=0.0293\,h^{0.725}\,m^{0.425}+0.0604$$

(5) $$C_{d}=4.45\,m^{-0.45}$$

Where h is the subject height and m is the body mass.

The effective surface area (ACd) was computed as the multiplication between A and Cd. ACd has been appointed as one of the most accurate variables to characterize the cyclist aerodynamics (26).

### Mechanical power

The total mechanical power was assessed by a Garmin Edge 510 (2 pieces because of battery capacity) for the old WR; whereas, for the new WR, a Garmin Edge 530 (permanent charging provided by a power bank) and the Kurbel power2max were used as a power meter in both attempts. These devices are validated and reliable (27, 28). Normalized power per kilogram was used to present the estimated cyclist mean total power. However, it is important to note that the cyclist’s total mechanical power (P_*TOT*_: Equation 4) is dependent on the sum of power to overcome drag (P_*d*_), power of bearing friction (P_*WB*_), power of the rolling resistance (P_*RR*_), Changes in Potential Energy (P_*PE*_), changes in kinetic energy (P_*KE*_) and the chain efficiency factor (E_*C*_), typically assumed as 0.976 (16):


(6) $$P_{T\,O\,T}=P_{d}+P_{W\,B}+P_{R\,R}+P\,P_{E}+P_{K\,E}/E_{C}$$

### Mechanical efficiency

The gross mechanical efficiency (GE) for both WRs was estimated based on the ratio between the total work (Wext) and the total energy expenditure (Etot) (18).


(7) $$G\,E=\frac{W_{e\,x\,t}}{E_{t\,o\,t}}$$

The Wext was obtained by converting of W to Kcal/h (1 W = 0.86 Kcal/h) and then calculating it for 24 h. The Etot were 27,500 Kcal (23,500 kcal + ca. 4,000 kcal basal metabolic rate) for the new WR and 25,900 (21,900 kcal + ca. 4,000 kcal basal metabolic rate) for the old WR.

### Physiological demands

The result of the HR monitoring in the two WRs was used to assess the physiological demands. The Garmin Edge 510 (2 pieces because of battery capacity) was used in the old WR and the Garmin Edge 530 (permanent charging provided by a power bank) in the new one to monitor the HR and to quantify the physiological demands (cardiovascular intensity).

### Statistical analysis

Descriptive information was presented in mean ± standard deviation (SD). The normality of the distribution was tested. Considering the approach of the present study, we conducted a Spearman correlation to verify the relationship between speed and distance during the event for each WR. Race time was divided into time intervals (0–6 h; > 6–12 h; > 12–18 h; > 18–24 h) to compare the performance between old and new WR. However, the time intervals were only approximately the same in the two records. An independent sample Man-Whitney test was used to compare covered distance and speed differences between the two WRs. The effect size was presented in crude values differences (Δ). The *t*-test comparisons between the HR variations by the time intervals between the two world records were made, and Cohen d effect sizes were assessed (without effect if *d* < 0.2, moderate effect if 0.2 ≤ *d* ≤ 0.8 and large effect if *d* > 0.8). The relationship between power and laps was tested through the Spearman correlation (rho) for both WRs. GraphPad Prism (version 5.0), SPSS 26 and JASP (version 0.14.1.0) were used for all statistical and graphical analyses, adopting a significance level of *p* < 0.05.

## Outcomes

### Pacing

**FIGURE 2:**
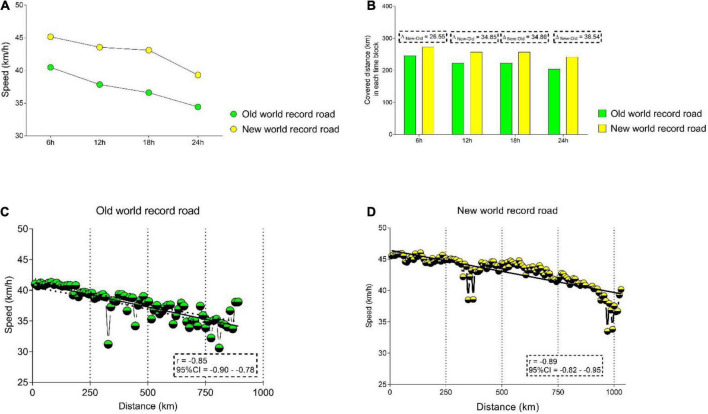
Performance differences between the two records, by time intervals. **(A)** Speed (km/h) comparisons for both records in each time interval; **(B)** covered distance (km) comparison for both records in each time interval; **(C)** relationship between speed (km/h) and distance covered (km) in old word record; **(D)** relationship between speed (km/h) and distance covered (km) in new word record.

Descriptive analysis and speed differences are presented in Figure 2. In the mean, the highest speed was shown in the new WR (42.97 ± 2.46 km/h). During the entire race, the speed of the new WR was higher compared to the old WR, but the highest speed difference was observed in the time block “ > 12–18 h,” where the athlete presented a mean speed of 43.09 km/h in new WR, against 36.59 km/h in the old WR (Δ = 6.50).

Differences in the covered distance (by each block) between the new and old WR are presented in Figure 2B. The highest difference in the covered distance was shown in the last part of cycling (Δ = 38.54). The relationship between speed and distance is presented in Figure 2C. For both WRs, a strong negative relationship was confirmed (ρ = –0.85; ρ = –0.89, in the old and new WR, respectively); that is, an increment in the distance is linked with a decrease in mean speed. A visual comparison between the two WRs shows a reduction in speed at approximately km 270.

**FIGURE 3:**
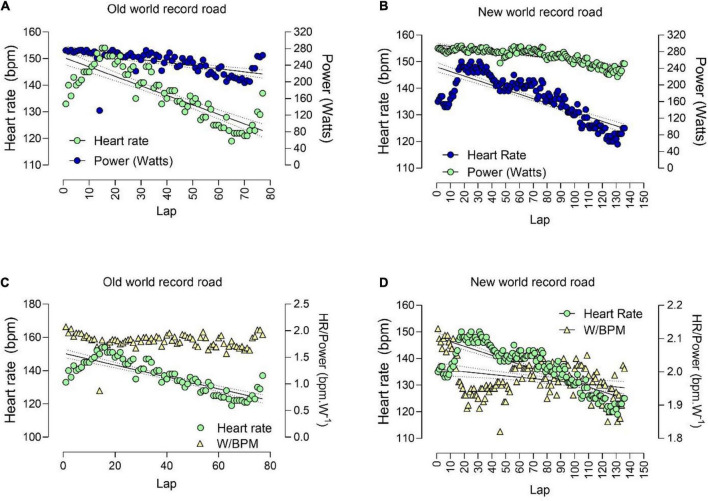
Relationship between power, heart rate and ratio between power and heart rate, for laps over time. **(A)** Relationship between power (watts), heart rate (bpm) and laps in old world record; **(B)** relationship between power (watts), heart rate (bpm) and laps in new world record; **(C)** heart rate (bpm) and ratio between power and heart rate (W/bmp) in old world record; **(D)** heart rate (bpm) and ratio between power and heart rate (W/bmp) in new world record.

Furthermore, we observed a higher variability in speed in the old WR, while in the new WR the speed mainly was stable. Speed explained approximately 72, and 79% of the performance variance in the two WRs, respectively. Figure 2D presents the Spearman correlation results for power (watts) across races. For both WR, a significant decrease in power is shown. For the new WR (B), the athlete started with a higher power and maintained it most of the time. A similar pattern was shown for HR in both WRs, except for the increment in HR in the final of the race in the old WR (A). Average HR for the first record was the same for both WRs (old WR, 136.36 ± 8.36 bpm; new WR, 136.57 ± 9.69 bpm), but the lowest and highest values were different (old WR, 119–150 bpm; new WR, 119–154 bpm) (Figure 3).

### Aerodynamics

The ACd was lower in the new WR (0.22066 m^2^) than in the old one (0.22121 m^2^). The drag difference between the new and old WR was 20% for drag and 0.25% for ACd. The measured mean total mechanical power was 275 W for the new and 254 W for the old WR, which is a difference of about 8%. These values suggest a higher capacity to produce power. The Power to overcome drag (P_*d*_) was 31% higher for the new WR than for the old WR. The Pd was estimated at 225.69 W for the new and 156.64 W for the old WR, respectively. The estimated Pd represented 82% of the total power in the new WR, whereas, in the old WR, it represented about 62%. These values suggest an improvement in the cyclist’ aerodynamic profile in the new WR. The cyclist’s total mechanical power per body weight (W/Kg) was higher in the new WR (3.44 W/Kg) in comparison to the old WR (3.26 W/Kg). The differences showed an almost 5% higher capacity to produce muscle power.

### Mechanical efficiency

The cyclist presented higher gross mechanical efficiency for the new WR compared to the old WR. A difference of 1% was noted between the races (Table 1).

### Physiological demands

**FIGURE 4:**
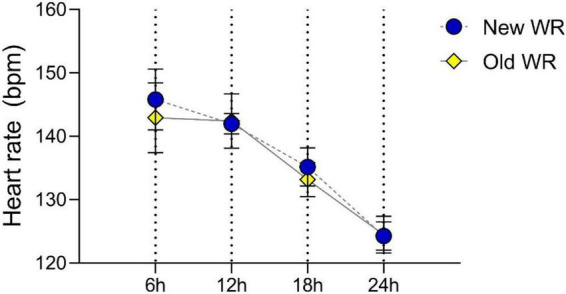
Heart rate (bpm) mean (standard deviation) for the two records by time intervals (6; 12 h; 18 and 24 h).

The comparison between the two WRs presented no statistically significant differences with small effect (*t* = 0.0672; *p* = 0.504; *d* = 0.078) (Figure 4). However, for the first 6 h (0–6 h) between the old (145.80 ± 5.88 bpm) and new WR (139.45 ± 5.82 bpm), significant differences were noted with a large effect (*t* = 6.312; *p* < 0.001; *d* = 1.411). Between > 6 and 12 h, no significant differences with a small effect were observed between the two WRs’ heart rate (Old HR = 142.40 ± 5.06 bpm; New HR = 142.20 ± 2.46 bpm) variability (*t* = 0.228; *p* = 0.822; *d* = 0.051). Regarding the time between > 12 and 18 h, statistically significant differences with a large effect (*t* = 4.767; *p* < 0.001; *d* = 1.192) were noted between the old (133.19 ± 3.53 bpm) and the new record (137.63 ± 2.80 bpm). For the last 6 h (> 18–24 h) of the race no statistically significant differences were observed with a moderate effect (*T* = 1.134; *p* = 0.272; *d* = 0.260). In the last phase, the old record HR mean was 124.47 ( ± 4.13) and 125.84 ( ± 2.34) bpm.

## Discussion

The study’s most important finding was the difference in performance between the two WRs. This can be observed through the longer covered distance and the higher cycling speed since the beginning (he started the race at a higher cycling speed and was able to keep it across all the races) compared to the previous WR. One possible explanation for the change in performance can be related to the ideas underlying the “Marginal Gains” concept.

The “Marginal Gains” concept was initially proposed by the Great Britain Cycling Team, which won eight gold medals in the London 2012 Olympic Games. The approach states that athletes’ performance can be at least minimally improved in several domains, such as leadership, technology, morphological features, training characteristics, pacing strategy, aerodynamic, rest, nutritional habits, psychological traits, contextual features (29). Thus, improving each of these domains by at least one per cent, could lead to an overall improvement in cycling performance due to the sum of all of them.

For the new WR, the athlete made some improvements in technical specifications after the old WR. For example, eliminating the bottle holder, improving the aerodynamic position by elevating the handlebar, and optimizing a stream-lined position of the upper body, wearing a special dress and helmet for time-trial cycling, using a larger front chain wheel (from 50 to 58) with another bottom bracket, ceramic wheel bearings, and tires of 26′′. Considering that aerodynamic drag is one of the main factors that impair the performance in a cycling race, the adjustments made aiming to provide better aerodynamics during the race can be linked to the performance improvement (26). Regarding the estimations of ACd and drag, based on cyclist anthropometrics, the cyclist Acd was reduced by about 0.25% between the two races. However, reducing Acd may allow the cyclist to improve the velocity for the same amount of drag.

As for removing the bottle holder, no study was found comparing bicycles with and without bottle holders. However, some studies assessed the bottle holder design in cyclist’s drag (30, 31); additionally, the tube’s circular shape improves the aerodynamics (31). By removing the bottle holder, the cyclist was able to, in theory, reduce the pressure differences between the front and rear boundaries and so minimize the pressure drag (15). Minimizing the pressure differences in every part of the bicycle-cyclist system will reduce the total drag. However, studies assessing the bicycle-cyclist system aerodynamics with or without the bottle holder are required to explain this phenomenon better. Another strategy was improving the cyclist’s position (adopting a stream-lined position) by elevating the handlebar. The cyclist’s positions may affect the drag by about 24% at a 40 km/h (32). The adopted stream-lined position allowed the cyclist to reduce the drag area and the coefficient of drag (32–34), thus improving performance. Several previous studies recommended that the cyclists adopt a time trial position as much as possible during a race (32, 35) to improve performance and reduce the energy cost. The cyclist also adopted a time trial type helmet. A novel road aero type helmet imposed less than 17% drag at 40 km/h. This difference allowed the cyclist to save about 13% of the energy cost (35). Using a time trial type helmet may have a higher impact on cyclist total drag. At least, for a slower velocity (23 km/h), the time trial helmet had 30% less drag than a road helmet (36). A special dress also helped reduce the drag, especially the viscous drag. The viscous drag results from estimating an elite road cyclist’s mechanical power and energy cost wearing standard and aero helmets. The dragged fluid on the body forms the first layer, and the following layer of fluid is dragged to the previous layer (37). A special dress may contribute to the reduction of the cyclist’s friction. The viscous drag will also be reduced by reducing the surface friction (35, 38). However, viscous drag has a smaller contribution to cycling. The wheels used could minimize the bicycle-cyclist total drag, reducing space for air vents (minimizing pressure drag) (35). The wheels’ size and design influence the cyclist’s drag (39). Specific wheels might be chosen based on the bicycle-cyclist characteristics. The cyclists compete in different conditions, and the weather will affect their performance. Typically, in rainy conditions, the fluid (air) has a higher density; however, as we’re more elevated than the sea level, the air is rarefied, and its density is lower. Also, higher temperatures seem to reduce drag, affecting the fluid density. The drag (F_*d*_ = 0.5.A.C_*d*_.v^2^) is dependent on the bicycle-cyclist system (area and coefficient of drag; A and C_*d*_), air density (ρ), and velocity (v^2^). Changing the fluid (ρ: air) density will affect the drag and performance will vary. In this case, competing at 650 above sea level might be a positive condition to reduce drag. However, the rainy periods might negatively affect (increasing the drag). For that reason, dry clothes were important to minimize the viscous drag. The weather conditions and technical strategies seemed to have helped the cyclist reduce drag, mechanical power, and energy cost during the race (32, 35). Altogether, the different choices allowed the cyclist to minimize the speed variations and break the world record. The smaller speed variations possibly allowed the cyclist to maintain the self-selected pace, minimizing the effort. The drag is dependent of the squared velocity (Fd = 0.5.p.A.Cd.v^2^). For the new WR, the cyclist was able to increase the mean velocity (42.75 km/h vs. 37.34 km/h) and so the drag will also expressively increase. The drag is an important variable, especially for training prescription regarding intensities. However, this is also a reason why ACd is a more accurate variable to quantify aerodynamics. Moreover, for the old record, if the cyclist was able to present the ACd of the new world record, the drag will reduce 0.25%. That may allow to increase the velocity from 37.34 to 37.38 km/h, improving the time at 1,000 km in 28.66 s. If the ACd did not improved between WR, the new WR would have a mean speed of 42.69 km/h (vs. 42.75 km/h); after 24 h, the total distance would be 1024.56 (less 1.64 km/h). Also, for the covered distance in the new WR (1026.2 km), for the old ACd, the cyclist would require more 32.88 s to reach the 1026.2 km. Finally, the racing day was self-selected by the cyclist and did not taken into account the higher, altitudes and temperatures that may positively influence the aerodynamics (40).

As for the mechanical power, the cyclist produced a total mechanical power of 275 W for the new WR and 254 W in the old WR, improving 8%. The mechanical power is mainly dependent on drag. The drag represented 82% of the total mechanical power in the new WR and 61.62% of the old WR. That may suggest that power of bearing friction (PWB), power of the rolling resistance (PRR), Changes in Potential Energy (PPE), changes in kinetic energy (PKE) and the chain efficiency factor (EC) were minimized with the cyclist strategies and improvements. The mechanical power delivered by the cyclist was in agreement with some studies that estimated PTOT at different velocities. In a study comparing helmets at 11.11 m/s (typically the mean speed in tours), estimations of P_*TOT*_ were between 224.97 and 271.05 W (35). These values are below the estimated P_*TOT*_ in the present study. However, that can be explained by the power dependence on velocity and the velocity differences (more than 0.77 m/s in the new WR compared to the referenced study) between the cyclist of the present study and the literature (35). More studies support the estimated mechanical power for the cyclist in this case study. Vogt et al. (41) presented mechanical power values of professional cyclists between 190 and 392 W at 11.41 m/s (speed below the “new world record”). Grappe (42) reported values of 250 W at a mean speed of 11 m/s in the time-trial position and Forte et al. (32), values between 250 and 300 W between settings. Regarding the power per kilogram (W/Kg), the cyclist in the current study was able to increase 5% between the old and new WR (3.25 W/Kg vs. 3.44 W/Kg, respectively). The cyclist of the present study increased body mass, allowing him to improve their performance. It is important to note that despite the cyclist having an increased body mass, the increase in power was proportionally greater, ensuring greater W/kg. Typically, the cyclists try to reduce the body mass and maintain the mean maximal P_*TOT*_ intending to increase the power to weight ratio (43, 44). At least one study presented the power to weight ratio in the Tour the France for 4 h of race (45) and the values were between 3.7 and 4.9 W/Kg for the years 2008–2013. Additionally, a recent study reported power per weight ratio in male professional cyclists based on different categories and typologies. For training sessions of 240 min, the values varied from 3.83 and 4.63 W/Kg (46). Again, these values are slightly above the cyclist of this study. However, it is important to note that the cyclist in study race for 24 h. The ability to maintain such high values was diminished by fatigue. Other studies presented higher values, but the competition times were lower in comparison with the current study (44, 47).

The cyclist’s mechanical efficiency was computed as the ratio between Wext and Etot. The present study estimated the GE for the old WR as 20 and 21% for the new WR. The results allow us to speculate that the cyclist improved his efficiency by about 1%. The values agree with the literature, where the cyclist’s efficiency is near 20%, and the values vary between 18 and 25% (18, 48). Again, the adopted strategies for race, training experience and individual characteristics (i.e., anthropometrics) may justify the improvement in GE.

The physiological demands were similar between the two WR. However, the time interval analysis revealed a statistically significant difference for the first 6 h (0–6 h). This shows that the cyclist started the race at lower intensity in the new WR. However, between the interval > 12 and 18 h, significant differences were noted, where the cyclist increased the intensity and physiological demand. In the last 6 h (>18–24 h) the race intensity between the two WRs remains similar with a moderate effect and higher HR mean values for the new WR. This may be the result of the cyclist’s strategy to attain the new WR, where the cyclist monitors HR variations to regulate the physical and physiological demands (19, 20, 23). However, the literature lacks individual racing strategies based on HR variations. We also need to consider that the athlete gained experience and benefited from physiological adaptations as a result of permanent exercise focused on performance. An improvement in experience might have helped to improve athletic performance. Recent studies showed that training intensity and competition was important (18, 49, 50). For ultra-marathoners, it has been shown that ultra-endurance mountain athletes competing in longer races have more experience and train harder than athletes competing in shorter distances (50). Successful finishers in the “TransEurope FootRace” 2009 showed that the extent of pre-race training in the last year before the race and personal best times for marathon and specific ultra-marathons have a high correlation to race performance (49). In ultra-triathletes competing in x-times the Ironman-distance triathlons, previous experience (i.e., fast personal best times of shorter races) seemed important in performance for longer ultra-triathlon races (51). Based on the power metrics, the cyclist could produce more power per kilogram (about 5% more). That can also be explained by the improved aerodynamics and experience/training. Overall, the cyclist was faster in the first 6 h of the new WR and was able to maintain relative speed in the next splits showing a more even pacing compared to the old WR. It is well known that pacing is performance-related with faster endurance and ultra-endurance athletes presenting more even pacing compared to their slower peers.

Another aspect to consider is motivation (52–54). It has been reported that ultra-marathoners showed a different motivation compared to runners of shorter race distances (55) and younger ultra-marathoners (54).

### Limitations

The main limitations of the present case report include the lack of control for variables that are directly related to cycling performance. For example, a recent review highlighted the multi-dimensional characteristics of performance, which involve the interaction between a plethora of domains that can explain the performance. The authors highlighted those as being: the individual dimension, the tactical dimension, the strategic dimension and the global dimension (29). Furthermore, in a previous case report, authors discussed the tactical and strategic dimensions using data of the same athlete (24). However, some information regarding individual characteristics can be useful in understanding the progress made between the records, such as the physiological index, diet habits, motivation, and cognitive traits. It important to note that this manuscript presented the world record monitored performance and so, laboratory tests were not performed (VO_2_max, lactate, etc.).

## Conclusion

Changes in strategies (i.e., technical changes, aero position) adopted in the new WR may explain the performance improvement and differences between the two WRs. The marginal gains concept can be an important insight for future athletes and coaches to consider during their long-term planning. The lack of data about the body composition and physical fitness of the cyclist can also be considered as a limitation.

## Athlete perspective

From an athlete’s perspective, the ultra-cyclist of the present study should be further encouraged to apply his actual training strategies (taking advantage of both scientific and technological advances) in the near future to improve his performance successfully. The changes he adopted during his preparation and the record might be adopted as a paradigm to follow by elite sports performance athletes. The athlete started working on air resistance values, performance, calorie consumption, rolling resistance and the best altitude and course to select 1 year before the event. But in the end, he found that he had the best day to break the record.^12^^,^^13^

## Data availability statement

The raw data supporting the conclusions of this article will be made available by the authors, without undue reservation.

## Ethics statement

Written informed consent was obtained from the individual(s) for the publication of any potentially identifiable images or data included in this article.

## Author contributions

BK and PF drafted the manuscript. MT and PF performed the data analyses. KW and MT helped in drafting the manuscript. All authors wrote and approved the final version.

## Footnotes

1
https://ultracycling.com/archive/stanislav-verstovsek-24-hour-road-record-attempt-oct-2-3-2020

2
https://ultracycling.com/archive/christoph-strasser-24-hour-road-attempt-july-16-17-2021

3
www.christophstrasser.at/

4
https://austria-forum.org/af/AustriaWiki/Fliegerhorst_Hinterstoisser

5
www.christophstrasser.at/24h_road_rekord_2021/

6
https://bikeboard.at/Board/Interview-Christoph-Strasser-1day1000k/-th264021

7
http://www.christophstrasser.at/24h_road_rekord_2021/detailansicht/aktuelles/mein-erstes-fazit-1day1000k

8
https://peeroton.com/dein-ziel/leistungssteigerung-erhalt/213/hi-end-endurance-energy-drink-professional-600g-pfirsich-christoph-strasser

9
https://nutrition.abbott/ch/de/product/ensure-plus#

10
www.panaceo.com/panaceo-energy-boost3-pulver-250-g

11
www.power2max.com

12
http://www.christophstrasser.at/aktuelles_live_newsletter/news/akt uelles_detailansicht/mein-erstes-fazit-1day1000k/

13
https://bikeboard.at/magazin/interview-christoph-strasser-1day100 0k-th9611
